# Local Anæsthesia

**Published:** 1896-05

**Authors:** 


ARTICLE IV.

### LOCAL ANESTHESIA.

Dr. Schleich gives the following three formulas :

(NO. 1.) STRONG SOLUTION.

For operations on inflamed or hyper-esthetic areas, as carbuncles, abscesses, where the pain of infiltration would be very great :

| | | | |
|---|---|---|---|
| R.—Muriate of Cocain, | - | gr. | 3 |
| Muriate of morphine, | - | gr. | 1-3 |
| Chlorid of sodium | - | gr. | 3 |
| Distilled water enough to make, | | oz. | 3 |

Sterilize by adding 3 drops of a 5 per cent. solution of carbolid acid.

(NO. 2.) STANDARD SOLUTION.

For surgical operations in general, and especially deep ones :

| | | | |
|---|---|---|---|
| R.—Muriate of cocain, | - | gr. | 1½ |
| Muriate of morphin, | - | gr. | 1-3 |
| Chlorid of sodium | - | gr. | 3 |
| Distilled water enough to make, | | oz. | 3 |

Sterilize by adding 3 drops of a 5 per cent. solution of carbolic acid.

(No 3.) WEAK SOLUTION.

For operations on superficial areas not rendered hyper-sensitive by inflammation, as in the removal of a nevus, etc. :

| | | | |
|---|---|---|---|
| R.—Muriate of cocain, | - | gr. | 1-6 |
| Muriate of morphin, | - | gr. | 1-12 |
| Chlorid of sodium, | - | gr. | 3 |
| Distilled water enough to make, | | oz. | 3 |

Sterilize by adding 3 drops of a 5 per cent. solution of carbolic acid.

The cocain renders the tissues anesthetic at once, though that is evanescent, yet the morphin serves to hold the effect, and the edema and pressure caused by the large injection make the anesthesia complete for about twenty to thirty minutes.

It is necessary to have a large hypodermic syringe for this purpose. Even the pain of the initial puncture of the large needle may be prevented by previously injecting a few drops of the ordinary 4 per cent. solution with a small syringe. As cold helps to produce and prolong the anesthesia, it is better that the solution should be made ice cold, if convenient. Dr. Bransford Lewis, of St. Louis, in an article in the *Medical Standard*, giving his experience in the use of this method, says:

"Every tissue of the body, without exception (skin, muscle, gland, mucous membrane, nerves, etc.), becomes insensible to pain when infiltrated in the manner described. This obtains for bone and the hard structures as well as the soft. Bone is reached either through infiltrating its periosteum or by injecting into the medulla. Nerve trunks are anesthetized separately, first by applying 5 per cent. carbolic acid solution and then through this inserting the needle and fluid.

Only the infiltrated, artificially edematous tissue is anesthetic, the tissues just outside retain normal acuteness or sensibility. Consequently, in the course of an operation, with absorption of the infiltrated fluid, it is necessary to renew the injections or extend their area coincidently with the operative field.

With the proper fluid anesthesia ensues immediately on its being introduced into the tissues, and lapse of time is not requisite for developing insensibility. This again is in marked contrast to the effect of the older methods of producing anesthesia. Its advantage is great.

Anemia resulting from the method, there will be less bleeding (oozing) than under ordinary circumstances. Distortion of the tissue from the infiltrated fluid does not cause any especially increased difficulty in securing and tying or twisting bleeding vessels. Nevertheless, in operating in deeper structures, care must be taken to avoid. risk of piercing blood-vessels, nerves, etc."

This is the method which we mentioned some time ago, stating that Professor Parvin had allowed a demonstration of it to be made on himself before the Philadelphia County Medical Society, allowing a deep cut to be made in his arm and sewed up under this infiltration method of producing local anesthesia.

It appears that we have witnessed another of those great discoveries which mark the rapid progress of medical and surgical science. In the early years of this century patients had to be held by several assistants while the operation was performed, while their agonizing screams gave additional distress to all around. Now they can read a newspaper while it is going on, or watch it with the interest which they may naturally be supposed to take in it.—*Med. World.*

